# Can rectal MRI and endorectal ultrasound accurately predict the complete response to neoadjuvant immunotherapy for rectal cancer?

**DOI:** 10.1093/gastro/goae027

**Published:** 2024-04-08

**Authors:** Menglan Zhai, Zhenyu Lin, Haihong Wang, Jinru Yang, Mingjie Li, Xin Li, Lan Zhang, Tao Zhang

**Affiliations:** Cancer Center, Union Hospital, Tongji Medical College, Huazhong University of Science and Technology, Wuhan, Hubei, P. R. China; Hubei Key Laboratory of Precision Radiation Oncology, Wuhan, Hubei, P. R. China; Institute of Radiation Oncology, Union Hospital, Tongji Medical College, Huazhong University of Science and Technology, Wuhan, Hubei, P. R. China; Cancer Center, Union Hospital, Tongji Medical College, Huazhong University of Science and Technology, Wuhan, Hubei, P. R. China; Hubei Key Laboratory of Precision Radiation Oncology, Wuhan, Hubei, P. R. China; Institute of Radiation Oncology, Union Hospital, Tongji Medical College, Huazhong University of Science and Technology, Wuhan, Hubei, P. R. China; Cancer Center, Union Hospital, Tongji Medical College, Huazhong University of Science and Technology, Wuhan, Hubei, P. R. China; Hubei Key Laboratory of Precision Radiation Oncology, Wuhan, Hubei, P. R. China; Cancer Center, Union Hospital, Tongji Medical College, Huazhong University of Science and Technology, Wuhan, Hubei, P. R. China; Institute of Radiation Oncology, Union Hospital, Tongji Medical College, Huazhong University of Science and Technology, Wuhan, Hubei, P. R. China; Cancer Center, Union Hospital, Tongji Medical College, Huazhong University of Science and Technology, Wuhan, Hubei, P. R. China; Institute of Radiation Oncology, Union Hospital, Tongji Medical College, Huazhong University of Science and Technology, Wuhan, Hubei, P. R. China; Department of Radiology, Union Hospital, Tongji Medical College, Huazhong University of Science and Technology, Wuhan, Hubei, P. R. China; Department of Radiology, Union Hospital, Tongji Medical College, Huazhong University of Science and Technology, Wuhan, Hubei, P. R. China; Cancer Center, Union Hospital, Tongji Medical College, Huazhong University of Science and Technology, Wuhan, Hubei, P. R. China; Hubei Key Laboratory of Precision Radiation Oncology, Wuhan, Hubei, P. R. China; Institute of Radiation Oncology, Union Hospital, Tongji Medical College, Huazhong University of Science and Technology, Wuhan, Hubei, P. R. China

**Keywords:** locally advanced rectal cancer, clinical complete response, rectal MRI, endorectal ultrasound, immunotherapy

## Abstract

**Background:**

Standardized assessments of clinical complete response (cCR) to neoadjuvant chemoradiotherapy (nCRT) for rectal cancer have been established, but their utility and accuracy remain unclear. This study aimed to evaluate the clinical diagnostic value of rectal magnetic resonance imaging (MRI) and endorectal ultrasonography (ERUS) for the determination of cCRs after neoadjuvant immunotherapy and to investigate the concordance between cCR and pathological complete response (pCR).

**Methods:**

Ninety-four patients with rectal cancer treated with neoadjuvant radiotherapy with or without immunotherapy were included. The sensitivity, specificity, and accuracy of each evaluation method were calculated.

**Results:**

Combined MRI and ERUS assessments found cCR in seven of the 94 patients in our cohort. In the non-immunotherapy group, the sensitivity, specificity, and accuracy of MRI for diagnosing cCR were 50.0%, 85.2%, and 77.1%, respectively, whereas those of ERUS were 50.0%, 92.6%, and 82.9%, respectively; those of combined MRI and ERUS were 25.0%, 96.3%, and 87.5%, respectively. In the immunotherapy group, the sensitivity, specificity, and accuracy with which MRI identified CR were 51.7%, 76.7%, and 64.4%, respectively; those of ERUS were 13.8%, 90.0%, and 52.5%, respectively, and those of combined MRI and ERUS were 10.3%, 96.7%, and 54.2%, respectively. We also found that 32 of 37 patients with pCR did not meet the cCR evaluation criteria. Of these pCR patients, 78.4% (29/37) received immunotherapy. In the entire cohort, there were five pCRs among the seven cCRs. Of the four cCRs that occurred in the immunotherapy group, three were pCRs.

**Conclusions:**

Rectal MRI and/or ERUS did not provide sufficiently accurate assessments of cCR in patients with rectal cancer receiving neoadjuvant therapy, especially immunotherapy, and cCR did not predict pCR.

## Introduction

Neoadjuvant chemoradiotherapy (nCRT) combined with total mesorectal excision is the current standard treatment mode for patients with middle and low locally advanced rectal cancer (mlLARC) [1]. In recent years, the rise of immunotherapy has dramatically changed the treatment landscape for many malignant solid tumor types, including rectal cancer [2]. Radiotherapy can act as an in-situ vaccine, and its combination with immunotherapy can exert synergistic antitumor effects [3, 4]. The pathological complete response (pCR) rate to radical surgery after conventional nCRT is approximately 15%–20% [5, 6]. Several studies have shown that adding immunotherapy to radiotherapy or chemoradiotherapy greatly increases pCR rates [7, 8]. Short-course radiotherapy (SCRT) with sequential immunotherapy increased the pCR rate to 48.1% in the UNION phase II clinical trial conducted at our center [9]. Some patients achieved clinical complete response (cCR) after receiving neoadjuvant therapy. For these patients, a watch-and-wait strategy rather than conventional radical surgery can be considered to avoid potential postoperative complications [10]. However, when conventional nCRT mode is used, the relationship between cCR and pCR is inconsistent. More than half of non-cCR patients are actually pCR [11], and nearly half of cCR patients are confirmed as non-pCR by postoperative histopathology [12]. Rectal magnetic resonance imaging (MRI) and endorectal ultrasonography (ERUS) are widely used to evaluate cCR in patients with mlLARC after nCRT. Compared with conventional chemoradiotherapy, the application of immunotherapy can induce more immune cell infiltration, necrosis, fibrosis, or edema in the tumor microenvironment [13–15]. Therefore, we suspect that rectal MRI and ERUS alone may not be sufficient to assess cCR in mlLARC patients receiving immunotherapy. Therefore, in this study, we evaluated cCR using preoperative ERUS and rectal MRI and explored the consistency between these cCR results and pCR findings obtained by postoperative histopathology with and without neoadjuvant immunotherapy.

## Patients and materials

**Figure 1.**
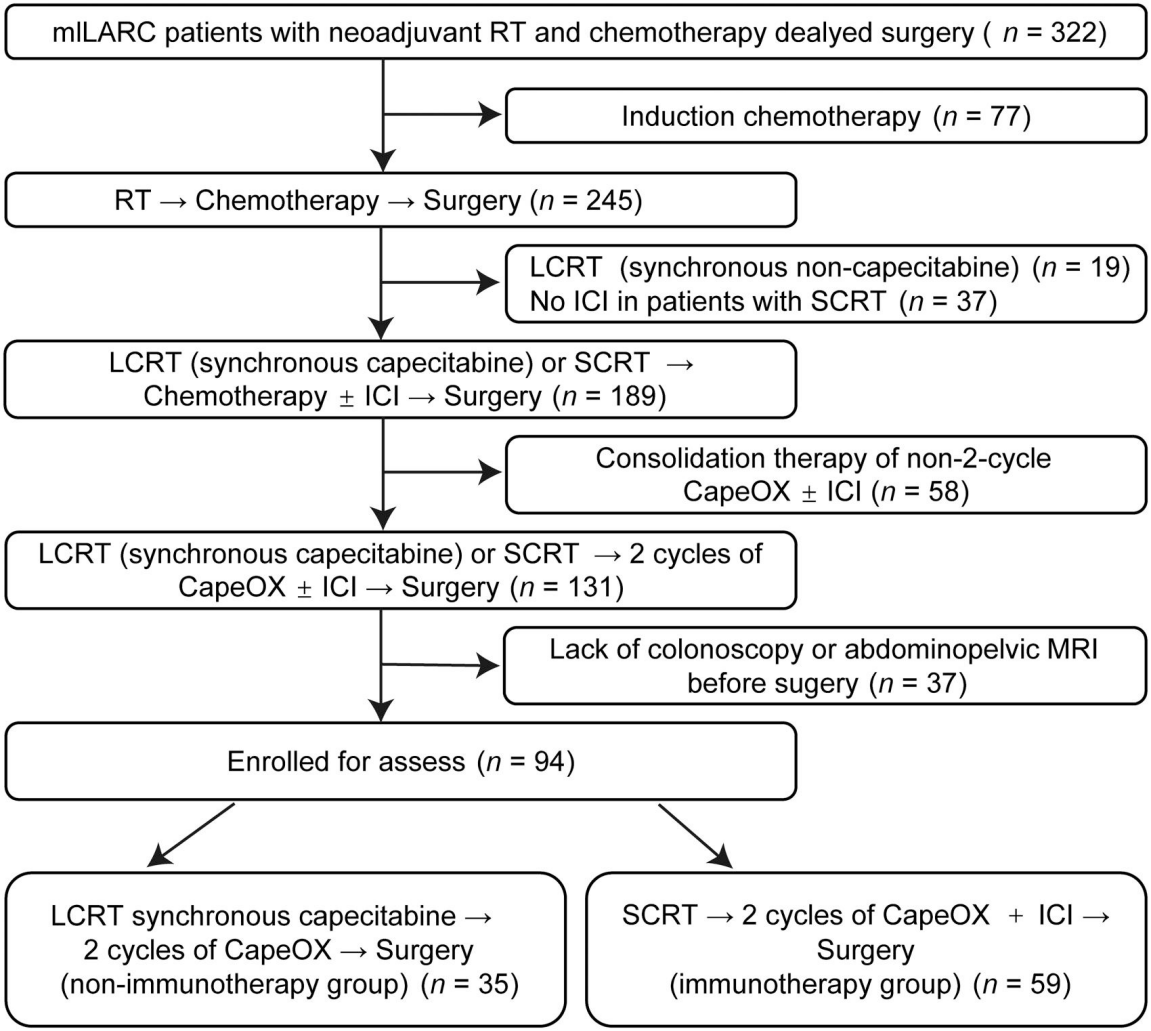
Flow chart of the eligible patient screen. mlLARC = middle and low locally advanced rectal cancer, RT = radiotherapy, LCRT = long-course chemoradiotherapy, ICI = immune checkpoint inhibitor, SCRT = short-course radiotherapy, MRI = magnetic resonance imaging.

The medical records of mlLARC patients who underwent nCRT delayed surgery at Union Hospital, Tongji Medical College, Huazhong University of Science and Technology (Wuhan, China) between July 2015 and June 2022 were collected retrospectively (including the data of patients with neoadjuvant immunotherapy for UNION phase II rectal cancer in our research institution). Inclusion criteria were as follows: (1) histologically confirmed adenocarcinoma within 10 cm from the anal verge; (2) clinical stage of cT3-4N0 or N+ with no evidence of distant metastases; (3) receiving either long-course chemoradiotherapy (LCRT) or SCRT sequential 2 cycles of CapeOX (oxaliplatin + capecitabine) with or without immune checkpoint inhibitor (ICI) therapy, and (4) preoperative complete ERUS and rectal MRI. The detailed screening process is shown in Figure 1. The study has been approved by the Institutional Review Board of Union Hospital, Tongji Medical College, Huazhong University of Science and Technology. The requirement for informed consent was waived owing to the retrospective nature of the study.

### Neoadjuvant therapy and surgery

Patients who met the inclusion criteria received preoperative radiotherapy (LCRT or SCRT). LCRT comprised a total of 45–50.4 Gy in 25–28 fractions administered 5 days per week for the duration of radiotherapy concomitantly with oral capecitabine 825 mg/m^2^/day chemotherapy. SCRT refers to five fractions of radiotherapy over five days at a total dose of 25 Gy. During the interval between radiotherapy and surgery, the patients were given 2 cycles of CapeOX (oxaliplatin 130 mg/m^2^ intravenously on day 1; capecitabine 1,000 mg/m^2^ twice per day on days 1–14) chemotherapy with or without camrelizumab (200 mg intravenously on day 1, only in the SCRT group). All patients underwent TME operation including Dixon, Miles, and Hartmann procedures after completion of neoadjuvant therapy. ERUS and rectal MRI were required in all patients before the operation.

### Tumor assessment

Staging of rectal cancer was performed according to the Union for International Cancer Control/American Joint Committee of Cancer (UICC/AJCC) v.7.0/8.0. Baseline and preoperative examinations included thoracoabdominal and pelvic computed tomography (CT), ERUS, and rectal MRI. Serum levels of carcinoembryonic antigen (CEA) were also tested. The cCR assessment criteria were as follows [16]: (a) No residual tumor, white scar with or without telangiectasia, and no ulcers or nodules on endoscopic examination; (b) MRI T2-weighted images show only dark T2 signals with no intermediate T2 signals and no visible lymph nodes; and (c) No visible tumor in diffusion-weighted images on b800-b1000 signals with or without any signal or a low signal on apparent diffusion coefficient maps. We defined pCR (ypT0N0) as the absence of residual tumor in resected specimens during the histological examination [17].

Two gastrointestinal radiologists (with 13 and 28 years of experience in rectal imaging) blinded to the histopathologic outcomes independently reviewed the baseline and preoperative MRI images. In instances in which their conclusions were inconsistent, the images were evaluated by a third radiologist. All ERUS was performed by endoscopy physicians. The final assessment of cCR for each case was decided by the attending physician based on their clinical judgment using the above assessment criteria.

### Statistical analysis

The data were analyzed by SPSS 25.00 software (IBM Corporation, Chicago, IL, USA). Sensitivity, specificity, accuracy, and the Youden index were used to assess the diagnostic efficacy of MRI, ERUS, and serum CEA levels alone and in combination. In joint analysis, the three examinations should meet the diagnostic criteria of cCR at the same time. The measurement data are expressed as mean ± standard deviation (SD) and the count data are described as case numbers or percentages. Comparisons between groups were made by *t*-test and Chi-square test with a test level of α = 0.05 (two sides). The *P* values of <0.05 were statistically significant.

## Results

### Patient characteristics

This study enrolled 94 patients with mlLARC, including 59 men (62.8%) and 35 women, with a mean age of 56.2 ± 9.0 years. Among the 94 patients, 37 (39.4%) were confirmed pCR after neoadjuvant therapy (non-immunotherapy or immunotherapy) by histopathology. The immunotherapy group had a significantly higher pCR rate than the nonimmunotherapy group (49.2% vs 22.9%, *P *=* *0.012). The interval between the end of radiotherapy and surgery was longer in the non-immunotherapy group than in the immunotherapy group (Table 1).

**Table 1.** Characteristics of the 94 patients at baseline

Characteristic	Non-immunotherapy group (*n *=* *35)	Immunotherapy group (*n *=* *59)	*P* value
Gender, *n* (%)			0.181
Male	25 (71.4)	34 (57.6)	
Female	10 (28.6)	25 (42.4)	
Age, mean ± SD, years	57.9 ± 8.6	55.2 ± 9.2	0.171
Distance from tumor to the anal verge, *n* (%)			
0–5 cm	19 (54.3)	28 (47.5)	0.522
>5–10 cm	16 (45.7)	31 (52.5)	
Mismatch repair status, *n* (%)			
pMMR/MSS	33 (94.3)	59 (100.0)	-
dMMR/MSI-H	2 (5.7)	0 (0.0)	
The time from the end of radiotherapy to surgery, mean ± SD, days	73.2 ± 8.5	66.4 ± 12.5	0.006
Preoperative staging (TNM), *n* (%)			
MRI			0.147
cCR	8 (22.9)	22 (37.3)	
non-cCR, *n* (%)	27 (77.1)	37 (62.7)	
ERUS			0.474
cCR	6 (17.1)	7 (11.9)	
non-cCR	29 (82.9)	52 (88.1)	
MRI+ERUS, *n* (%)			1.000
cCR	3 (8.6)	4 (6.8)	
non-cCR	32 (91.4)	55 (93.2)	
Postoperative staging (TNM), *n* (%)			0.012
pCR	8 (22.9)	29 (49.2)	
non-pCR	27 (77.1)	30 (50.8)	

SD = standard deviation, pMMR/MSS = proficient mismatch repair/microsatellite stability = dMMR/MSI-H = deficient mismatch repair/microsatellite instability-high, MRI = magnetic resonance imaging, cCR = clinical complete response, pCR = pathological complete response, ERUS = endorectal ultrasound.

### Results of the different evaluation methods

In the non-immunotherapy group, eight patients were diagnosed with pCR after neoadjuvant therapy. Preoperatively, cCR was found in eight, six, and three patients evaluated by MRI alone, ERUS alone, and MRI and ERUS combined, respectively. In the immunotherapy group, the same preoperative evaluation methods showed that 29 patients achieved pCR, and 22, 7, and 4 patients met the cCR evaluation criteria (Table 2).

**Table 2.** Evaluation results of different inspection methods in the non-immunotherapy group and immunotherapy group

Postoperative pathology	N	MRI		ERUS		MRI + ERUS	
		cCR	non-cCR	cCR	non-cCR	cCR	non-cCR
Non-immunotherapy							
pCR	8	4	4	4	4	2	6
non-pCR	27	4	23	2	25	1	26
Immunotherapy							
pCR	29	15	14	4	25	3	26
non-pCR	30	7	23	3	27	1	29

MRI = magnetic resonance imaging, ERUS = endorectal ultrasound, cCR = clinical complete response, pCR = pathological complete response.

### Diagnostic efficacy

In the non-immunotherapy group, the specificity and accuracy of combined MRI/ERUS assessment of cCR were slightly higher than those for MRI and ERUS alone. In contrast, the sensitivity of combined assessment was slightly lower than that of MRI or ERUS alone. However, none of these differences were statistically significant. In the immunotherapy group, the sensitivity of combined assessment was significantly lower than that of MRI alone (10.3% vs 51.7%, *P *=* *0.009), whereas the specificity and accuracy were not significantly different between assessment methods (Table 3).

**Table 3.** Sensitivity and specificity of MRI and ERUS preoperative assessment methods

Parameter	Non-immunotherapy group			*P* value	Immunotherapy group			*P* value
	MRI	ERUS	MRI+ERUS		MRI	ERUS	MRI+ERUS	
Sensitivity, %	50	50	25	0.504	51.7	13.8	10.3	<0.001*
Specificity, %	85.2	92.6	96.3	0.335	76.7	90	96.7	0.055
Accuracy, %	77.1	82.9	87.5	0.836	64.4	52.5	54.2	0.371
Youden index	0.352	0.426	0.213	–	0.284	0.038	0.07	–

MRI = magnetic resonance imaging, ERUS = endorectal ultrasound, cCR = clinical complete response, pCR = pathological complete response.

* The difference between the three groups was derived from MRI versus MRI+ERUS.

In addition, the accuracy of ERUS alone (82.9% vs 52.5%, *P *=* *0.003) and that of MRI and ERUS combined (87.5% vs 54.2%, *P *=* *0.012) were higher in the non-immunotherapy group than in the immunotherapy group. Although the accuracy of assessments by MRI alone was higher in the non-immunotherapy group than in the immunotherapy group, the difference was not statistically significant (77.1% vs 64.4%, *P *=* *0.196). There was no statistical difference in sensitivity or specificity between the two groups for either individual or combined assessments (Table 3).

### Relationship between cCR and pCR

Seven patients in our sample were evaluated as having cCR by combined MRI and ERUS, of whom, 5 (71.4%) were confirmed to have pCR by postoperative histopathology. There were two pCRs in the three cCRs in the non-immunotherapy group and three pCRs in the four cCRs in the immunotherapy group. In the combined assessment, only five of 37 pCR patients met the cCR evaluation criteria, including two cCRs of eight pCRs in the non-immunotherapy group and three cCRs of 29 pCRs in the immunotherapy group (Table 2).

## Discussion

The assessment of treatment efficacy after neoadjuvant therapy is crucial for ongoing treatment planning and prognosis prediction. Digital rectal examination (DRE) after neoadjuvant therapy was not included in the final evaluation of this study because of their established inaccuracy and the lack of DRE documentation in most medical records. Our findings demonstrated that cCR is difficult to identify using MRI and ERUS, and that consistency between cCR and pCR is poor, making cCR an unreliable predictor of pCR.

This study assessed two groups of patients who received radiotherapy with or without immunotherapy. In the non-immunotherapy group, the sensitivity of the assessments was poor, whether using MRI alone, ERUS alone, or the two combined. The combined assessment did not significantly improve specificity. In the immunotherapy group, the sensitivity of the combined evaluation was lower than that of MRI alone or ERUS alone, and there was no difference in sensitivity between the latter two. The sensitivity of the combined assessment was lower in patients who received immunotherapy than in those who did not, but the difference was not statistically significant. The specificity was comparable between groups. For both individual and combined assessments, the accuracy was lower in the immunotherapy than in the non-immunotherapy group (but this difference was not statistically significant for MRI alone). Kye *et al*. [18] found that ERUS and MRI have low sensitivity and accuracy in predicting cCR but excellent specificity. A meta-analysis of 46 studies found that MRI predicted cCR with an overall sensitivity and specificity of 95% and 31%, respectively, whereas ERUS predicted cCR with an overall sensitivity and specificity of 97% and 30%, respectively [19]. Although no previous reports have specifically evaluated the efficacy of cCR in this patient population when they have received neoadjuvant immunotherapy, the existing data from non-immunotherapy samples combined with the results of the present study suggest that MRI and/or ERUS cannot effectively evaluate cCR, whether in those treated with conventional nCRT or those treated with combined radiotherapy and immunotherapy.

It is clear that using the current criteria, cCR cannot accurately predict pCR. In this study, 32 of 37 patients with pCR did not meet the criteria for cCR. Previous studies have also found poor consistency between cCR and pCR (Supplementary Table S1). Smith *et al*. [11] reviewed 31 patients with pCR rectal cancer and found that 19 did not meet the cCR criteria. In another study by the same authors, only 16 of 61 patients with pCR met the cCR criteria [20]. Compared with the results in previous studies, fewer patients with histopathologically confirmed pCR in this study were defined as having cCR. Conversely, 78.4% (29/37) of pCR patients received neoadjuvant SCRT with sequential immunotherapy. The time interval from the end of radiotherapy to surgery in these patients was shorter than that of traditional LCRT (non-immunotherapy) patients. There was a lag in the response to neoadjuvant immunotherapy; it took longer to achieve cCR in patients undergoing neoadjuvant immunotherapy than in those undergoing non-immunotherapy. Also, immune cell infiltration, necrosis, fibrosis, or edema could all interfere with the judgment of immunotherapy response, leading to an underestimation of the efficacy of immunotherapy [21, 22]. Further, because of the longer time interval required to reach cCR in patients receiving SCRT than in those receiving LCRT [23], the current national and international cCR criteria may not be applicable to the evaluation of efficacy in patients with neoadjuvant immunotherapy. In the present study, 32 of 87 non-cCR patients were pathologically diagnosed with pCR, including 26 of 55 non-cCR patients in the immunotherapy group and only six of the 32 non-cCR patients in the non-immunotherapy group. Balasuriya HD *et al*. [24] included LARC patients who received LCRT and found pCR in five of 36 non-cCR patients. It is apparent that, compared with non-immunotherapy, immunotherapy not only increases the proportion of pCRs but also reduces the proportion of cCRs using current evaluation methods. These findings indicate that conventional assessments of chemoradiotherapy responses may be unsuitable for the evaluation of responses to neoadjuvant immunotherapy. More efficient assessment of tumor regression after neoadjuvant therapy, accurate identification of cCR patients, and screening out of non-cCR pCR cases are challenges for clinicians and a research priority. In recent years, circulating tumor DNA (ctDNA) dynamic monitoring [25] and MRI radiomics [26, 27] have been proven valuable in predicting pCR in neoadjuvant therapy for rectal cancer. The combined use of multiple methods is likely to improve the accuracy of assessments.

There were also some limitations to this study. First, the size of our cohort was small. Therefore, our findings need to be verified with a larger sample. Second, this study lacks long-term follow-up data to compare the long-term prognosis of cCR versus non-cCR patients using the existing assessment modalities.

## Conclusions

Rectal MRI and/or ERUS did not accurately evaluate the cCR in the mlLARC patients receiving neoadjuvant radiotherapy and chemotherapy, especially those also undergoing combination immunotherapy. The consistency between cCR and pCR was found to be poor.

## Authors’ Contributions

M.Z., Z.L., and T.Z. conceived and designed the project. M.Z. and L.Z. collected the data. M.Z., L.Z., H.W., J.Y., M.L., and X.L. analyzed and interpreted the data. M.Z. and Z.L. drafted the manuscript. All authors read and approved the final manuscript.

## Supplementary Material

goae027_Supplementary_Data
