# Supplementary material for: Can rectal MRI and endorectal ultrasound accurately predict the complete response to neoadjuvant immunotherapy for rectal cancer?
Source: Gastroenterol Rep (Oxf). 2024 Apr 8;12:goae027. doi: 10.1093/gastro/goae027 (PMC11001488; doi:10.1093/gastro/goae027)
Supplement: goae027_Supplementary_Data [file goae027_supplementary_data.docx]

Table S1. Related research on the consistent rate of cCR and pCR

| Authors, Years |  | WW |  | SG |  | Distant  metastasis (%) | |  | Recurrence (%) | |  | Salvage  surgery (%) |  | Neoadjuvant therapy |  | Evaluate  time (weeks) |  | pCR/cCR (%) |  | DFS |  | OS |
| --- | --- | --- | --- | --- | --- | --- | --- | --- | --- | --- | --- | --- | --- | --- | --- | --- | --- | --- | --- | --- | --- | --- |
|  |  |  |  |  |  | WW | SG |  | WW | SG |  |  |  |  |  |  |  |  |  |  |  |  |
| Li J et al. 2015 [1] |  | 30 |  | 92 |  | 1 (3.3%) | 5 (5.4%) |  | 2 (6.7%) | 2 (2.2%) |  | 1 (3.3%) |  | 50 Gy/25 F, concurrent capecitabine |  | 8-10 weeks after RT |  | 81/92 (88%) |  | 5-year DFS: 90.0% vs 94.3% (*P* = 0.932) |  | 5-year OS:  100.0% vs 95.6%,  *P* = 0.912 |
| Martens MH et al.  2016 [2] |  | 61 |  | 15 |  | 3 (3.5%) | 2 (13.3%) |  | 12 (14.1%) | 3 (20%) |  | 12 (14.1%) |  | 50.4 Gy/28 F, concurrent capecitabine or 25 Gy/5 F |  | Initial assessment 8 weeks after RT, re-assessment after 3 months for those with near-cCR |  | 9/15 (60%)  (re-assessment) |  | NS |  | 3-year OS: 96.4% vs 97.1%, *P* = 0.87 |
| Han Z et al. 2022 [3] |  | 58 |  | 26 |  | 4 (6.9%) | 3 (11.5%) |  | 9 (15.5%) | 1 (3.8%) |  | 11 (19.0%) |  | 30 Gy/10 F concurrent capecitabine;  GTV 50.6 Gy and PTV 41.8 Gy/22 F, concurrent capecitabine |  | NS |  | 20/26 (76.9%) |  | close to 3 years (mean: 34 months)  81.1% vs 84.6%, *P* = 0.819 |  | close to 3 years  (mean: 34 months)  96.6% vs 92.3%, *P* = 0.403 |
| Wang QX  et al. 2021 [4] |  | 94 |  | 94 |  | 9 (9.6%) | 11 (11.7%) |  | 14 (14.9%) | 1 (1.1%) |  | 12 (85.7%) |  | FU-based chemoradiotherapy, most common dose 50 Gy; Concurrent chemoradiotherapy alone, or with induction and/or consolidation chemotherapy |  | NS |  | 48/94 (51.1%) |  | 3-year DMFS：  88% vs 89%, *P* = 0.874;  3-year non-regrowth DFS:  88% vs 89%, *P* = 0.869 |  | 3-year OS：99% vs 97%, *P* = 0.983 |
| Balasuriya HD et al. 2022 [5] |  | 15 |  | 39 |  | 0 | NS |  | 1 (9%) | NS |  | 1 (9%) |  | 45-54 Gy/30 F, Capecitabine/FU synchronization; consolidation chemotherapy (typically FOLFOX) |  | 8 weeks after radiotherapy |  | 4/5 (80%);  5 pCR out of 36 non-cCR |  | NS |  | NS |
| Smith FM et al. 2012 [6] |  | - |  | 220 |  | - | - |  | - | - |  | - |  | 45-50.4 Gy/28 F, concurrent 5-FU |  | NS |  | Of the 31 pCR cases, 19 were non-cCR and 12 were cCR |  | - |  | - |
| Smith FM et al. 2014 [7] |  | - |  | 238 |  | - | NS |  | - | NS |  | - |  | neoadjuvant chemoradiation  with a median dose of 54 Gy (range, 30.6-61 Gy) |  | NS |  | 16 cCR out of 61 pCR (ypT0) |  | - |  | - |

ERUS: endorectal ultrasound; cCR: clinical complete response; pCR: pathological complete response; WW: watch-and-wait; SG: Surgery; DFS: disease-free survival; OS: Overall Survival; GTV: gross tumor volume; PTV: plan target volume

References

1. Li J, Liu H, Yin J, et al. Wait-and-see or radical surgery for rectal cancer patients with a clinical complete response after neoadjuvant chemoradiotherapy: a cohort study. Oncotarget 2015;6:42354-61.
2. Martens MH, Maas M, Heijnen LA, et al. Long-term Outcome of an Organ Preservation Program After Neoadjuvant Treatment for Rectal Cancer. J Natl Cancer Inst 2016;**108**:djw171.
3. Han Z, Li M, Chen J, et al. Surgery may not benefit patients with locally advanced rectal cancer who achieved clinical complete response following neoadjuvant chemoradiotherapy. Asian J Surg 2022;**45**:97-104.
4. Wang QX, Zhang R, Xiao WW, et al. The watch-and-wait strategy versus surgical resection for rectal cancer patients with a clinical complete response after neoadjuvant chemoradiotherapy. Radiat Oncol 2021;**16**:16.
5. Balasuriya HD, Timon C, Entriken F, et al. Early results from implementation of a 'watch and wait' protocol for complete clinical response following chemoradiotherapy for rectal cancer. ANZ J Surg 2022;**92**:2961-7.
6. Smith FM, Chang KH, Sheahan K, et al. The surgical significance of residual mucosal abnormalities in rectal cancer following neoadjuvant chemoradiotherapy. Br J Surg 2012;**99**:993-1001.
7. Smith FM, Wiland H, Mace A, et al. Clinical criteria underestimate complete pathological response in rectal cancer treated with neoadjuvant chemoradiotherapy. Dis Colon Rectum 2014;**57**:311-5.
